# Feline Wharton’s jelly-derived mesenchymal stem cells as a feeder layer for oocytes maturation and embryos culture *in vitro*

**DOI:** 10.3389/fvets.2023.1252484

**Published:** 2023-10-06

**Authors:** Meriem Baouche, Małgorzata Ochota, Pascal Mermillod, Yann Locatelli, Wojciech Nizanski

**Affiliations:** ^1^Department of Reproduction and Clinic of Farm Animals, Wrocław University of Environmental and Life Sciences, Wrocław, Poland; ^2^Physiology of Reproduction and Behaviors (PRC), INRAE, CNRS, University of Tours, Tours, France; ^3^Museum National d’Histoire Naturelle, Réserve Zoologique de la Haute Touche, Obterre, France

**Keywords:** cat, oocytes, embryos, co-culture, mesenchymal stem cells, Wharton’s jelly

## Abstract

**Introduction:**

Due to their capacity to release growth factors and cytokines, co-culture using mesenchymal stem cells has been considered a good alternative to promoting the maturation of the oocytes and the embryo’s development quality *in vitro* in different mammalian species. In this regard, we investigated the effect of feline Wharton’s jelly MSCs as feeders layer in oocyte maturation—consequently, the development of resulting embryos in co-culture.

**Methods:**

Oocytes with dark cytoplasm and a few layers of cumulus cells were collected and subjected to *in vitro* maturation and embryo culture using commercial media with and without MSCs addition. The oocytes’ nuclear maturation and the degree of cumulus expansion in different groups were assessed after 24 h; the development of the embryo was evaluated every 12 h until day eight.

**Results:**

Although MSCs increased the proportion of cumulus cells oocytes exhibiting cumulus expansion, there were no significant differences in the percentage of matured oocytes (metaphase II) among the groups (*p* > 0.05). However, the embryo development differs significantly, with a higher cleavage, morula, and blastocyst percentage in oocytes matured with MSC co-culture conditions than in commercial media alone (*p* < 0.05). Also, we observed higher morula and blastocyst rates in the embryos co-cultured with MSCs during the *in vitro* culture (*p* > 0.05).

**Conclusion:**

Based on our results, the co-culture with MSCs during the oocyte maturation resulted in better embryo development, as well as the MSCs addition during embryo culture returned an increased number of morula and blastocysts. Further research is needed to fully understand and optimize the use of MSCs in oocyte maturation and embryo development.

## Introduction

Due to the decreasing number of wild felids, domestic cats (*Felis catus*) as a model for studying reproduction physiology and developing new assisted reproductive technologies (ART) are gaining more and more importance (1).

ART has been used for several years to preserve genetic material, circumvent problems of subfertility, improve male reproduction, and increase the reproductive results and number of offspring that a single female can obtain. The post-fertilization period is an essential step in culturing embryos *in vitro.* Therefore, it is crucial to carefully plan the culture conditions during this period to ensure proper embryonic development. Inadequate culture conditions can significantly impact embryonic homeostasis, leading to short-term changes in morphology, cell proliferation, and metabolism and resulting in apoptosis. Such changes can ultimately lead to a reduction in both the number and quality of the formed blastocysts.

After years of investigations, the basic nutritional requirements for oocytes and embryos have been established, guaranteeing successful *in vitro* development in horses (2), cattle (3), pigs (4), mice and humans (5). However, ART in a feline species is still not as efficient as in other animals. Unfortunately, the culture conditions dedicated for cat oocytes and embryos have not yet been adequately investigated, and on average, *in vitro*, only around 60% of cats’ oocytes reach the MII phase, and less than a half of the cleaved embryos become blastocyst (6). Furthermore, the low rate of embryo production from feline oocytes reflects the need for a better understanding of the developmental competence of feline oocytes and their specific requirements during *in vitro* maturation, fertilization and embryo development. Current ART procedures lack knowledge of the interaction of gametes with several components present in the reproductive system during the maturation of oocytes and early stages of embryo development. To mimic the *in vivo* complex microenvironment *in vitro*, recent advances used a co-culture of oocytes and embryos with oviduct epithelial cells, mesenchymal stem cells (MSCs), cumulus cells and extracellular vesicles (EVs) in the reproductive environment with the aim to obtain *in vitro* embryos with developmental levels similar to embryos derived *in vivo*.

MSCs possess multi-potentiality and properties of immunological and inflammatory regulation. Cell therapy based on their transplant is a promising approach, as these cells can develop into adipocytes, osteoblasts, chondrocytes, smooth muscle cells, and endothelial cells and can express many specific markers depending on the environmental conditions in which they are found (7). The most common sources of MSCs are of adult origins, such as bone marrow or adipose tissue, but their removal requires an invasive clinical procedure. Perinatal sources like an umbilical cord, mainly Wharton’s jelly, offer higher practical accessibility and good quality MSCs with a higher proliferation rate and more potent immunomodulatory properties (8). *In vitro*, MSCs can thus promote cell viability and angiogenesis by producing growth factors. They also stimulate the recruitment of endogenous stem cells by secreting chemokines and acting locally through cell–cell interactions based on receptor-ligand bonds or through nanotubes that transfer molecules and organelles (9).

MSCs’ properties make them a suitable candidate for improving the performance of *in vitro* production systems in mammalian species. In fact, many studies have used MSCs or their derived biomaterials in a co-culture system with oocytes and/or embryos, with most studies indicating improved embryo development (10). Furthermore, it has been shown that coculture with MSCs could rescue poor-quality embryos and enhance early embryonic development (11, 12). Additionally, coculture with MSCs has been observed to enhance the cytoplasmic and nuclear oocyte maturation *in vitro* (13, 14). Based on these findings, we hypothesise that feline Wharton’s Jelly-derived MSCs could improve oocyte maturation and embryo culture *in vitro*. In this regard, we aimed to evaluate the *in vitro* effect of fWJ-MSCs added as a feeder layer in the co-culture system during cats’ oocyte maturation and embryo development, in comparison to non-conditioned, commercial maturation and culture media.

## Materials and methods

All chemicals and reagents were purchased from Sigma Aldrich Poland unless stated otherwise. Ethical approval was not sought, as it is not required for studies carried out on cells obtained from tissues that were surgical waste (Decision No. 004/2021). Commercial media were used for the oocyte manipulation and maturation: IVF Bioscience, Bickland Industrial Park, Falmouth, United Kingdom.

### Mesenchymal stem cells isolation and characterization

MSCs were isolated and characterized, as mentioned in our previous study (8). Umbilical cords were collected from healthy queens (1.5–5 years old) after a normal birth and caesarean sections; the cells were obtained from Wharton’s jelly parts of the umbilical cord (fWJ-MSC—feline Wharton’s jelly mesenchymal stem cells) using collagenase type I at 0.02% in DMEM-LG. The cells were cultured in DMEM-LG containing 10% FBS and 1% PS at 37°C in humidity. Adherents’ cells were grown until reaching 80 to 90% confluence before each passage, and the medium was changed three times a week. Before fWJ-MSCs were used, the cells were identified and characterized based on their expansion rate, tri-lineage differentiation (adipocytes, chondrocytes, and osteoblasts), cell surface markers (CD44, CD90, CD34, and MHC II) and pluripotency genes expression (OCT4, SOX2, NANOG).

### Preparation of Wharton’s jelly mesenchymal stem cells

To use fWJ-MSCs as a feeder layer, the cells at passage 2 to 3 were seeded in four well plates at a density of 1 × 10^4^ cells/mL in DMEM-LG containing 10% FBS and 1% PS at 37°C in humidity until reaching 80% to 90% confluence, nonadherent cells were removed by washing twice with PBS. The adherent cells were inactivated with 10 μg/mL mitomycin C for 2 h to avoid nutrients competition. After a series of washes with PBS, the culture was maintained in DMEM-LG for 24 h before the oocytes or embryos were co-cultivated.

### Ovaries and oocytes collection

Ovaries were obtained from sexually matured domestic queens subjected to a routine ovariohysterectomy or ovariectomy at the University clinic and local veterinarians in Wroclaw. After surgical removal, ovaries were stored in PBS with 1% of Antibiotic Antimycotic Solution at 4°C for up to 24 h before the recovery of cumulus-oocyte complexes (COCs). COCs were collected by slicing ovaries with a #10 scalpel blade in an OPU medium. Isolated COCs were classified under a dissecting microscope. Only oocytes with evenly pigmented dark ooplasm and some layers of cumulus cells were selected for further procedures.

### *In vitro* maturation of cat oocytes

The selected COCs were placed in a four-well plate in 400 μL of plain bovine maturation medium (BoM) and plain equine maturation medium (EqM) or in the same medium with MSCs co-culture: BoM + MSCs or EqM + MSCs, under mineral oil and matured for 24 h at 38.5°C in 5% CO_2_ in the air with maximum humidity.

### *In vitro* fertilization

For *in vitro* fertilization, the oocytes were fertilized with frozen–thawed semen and cryopreserved according to the protocol described by Partyka et al. (15). Semen straw was thawed in a water bath at 37°C then washed in IVF medium followed by centrifugation at 35,000 rpm for 5 min. After 24 h of maturation, cumulus oocytes complex were washed in IVF medium; then incubated with 1 × 10^6^ motile spermatozoa/ml for 18 h in 400 μL of IVF medium under mineral oil at 38.5°C in 5% CO_2_ in the air with maximum humidity.

### Assessment of oocytes maturation

In order to establish the assessment of oocyte maturation after 24 h of IVM, all the cumulus cells were mechanically removed using a glass pipette overheated and pulled to achieve the diameters of approximately 165 μm, slightly larger than the oocyte. Oocytes were aspirated and blown out repeatedly until most cumulus cells were removed. After most of the cumulus cells were removed, the oocytes were washed twice and fixed with 4% formaldehyde for 15 min flowed by washing in PBS and then incubated in DAPI stain solution for 10 min in the dark and mounted on glass slides in drops of Vectashield (Vector Laboratories, Ltd. United Kingdom). The nuclear state of the stained oocytes was assessed under a fluorescence microscope (Olympus IX73) at 360 excitations and 450 nm emission. Oocytes with distinct polar body or two separate and bright chromatin spots were classified as entering the MII stage.

### Embryo culture and assessment of the embryo development

After fertilization, presumptive zygotes were washed and transferred to a new plate in a droplet of 50 μL of either BoM or EqM medium or co-culture BoM + MSCs, EqM + MSCs medium (depending on the part of the experiment) covered with mineral oil and incubated at 38.5°C in 5% CO_2_ in the air with maximum humidity for up to 8 days. To assess embryo development, morphological changes were evaluated and noted every 8 to 12 h. The subsequent developmental stages were noted for each group, and the blastocyst formation was recorded.

### Study design

#### Experiment 1: the effect of the co-culture with MSCs on the oocyte maturation and cumulus cell expansion

This experiment evaluated nuclear maturation and cumulus cell expansion. Oocytes were matured in 400 μL of IVF medium under mineral oil at 38.5°C in 5% CO_2_ in the air with maximum humidity. In total, 180 oocytes were used in this part of the study, and three independent replicates of 15 oocytes per experimental group were carried out. Study groups were as follows:

- Maturation in BoM (*n* = 45 oocytes).
- Maturation in EqM (*n* = 45 oocytes).
- Maturation in BoM + MSCs (*n* = 45 oocytes).
- Maturation in EqM + MSCs (*n* = 45 oocytes).

The degree of nuclear maturation was analyzed after 24 h.

#### Assessment of cumulus cells expansion

**Figure 1:**
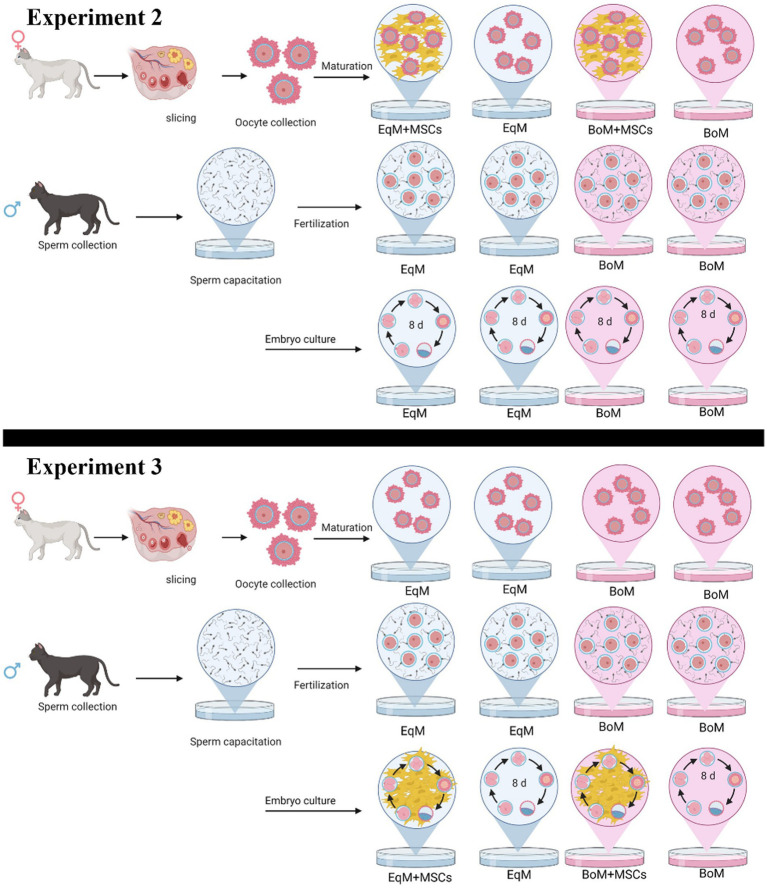
Study design of the experiment 2: the effect of the co-culture with MSC during oocyte maturation on the subsequent embryo development. Oocytes were matured and embryos cultured in four groups: EqM + MSCs/EqM: maturation in EqM + MSCs/embryo culture in EqM (*n* = 109). EqM/EqM: maturation in EqM/embryo culture in EqM (*n* = 109). BoM + MSCs/BoM: maturation in BoM + MSCs/embryo culture in BoM (*n* = 124). BoM/BoM: maturation in BoM/embryo culture in BoM (*n* = 103) and experiment 3: The effect of MSC addition during embryo development. Oocytes were matured, and embryo culture was carried out in four groups: EqM/EqM + MSCs: maturation in EqM/embryo culture in EqM + MSCs (*n* = 142). EqM/EqM: maturation in EqM/embryo culture in EqM (*n* = 109). BoM/BoM + MSCs: maturation in BoM/embryo culture in BOM + MSCs (*n* = 132). BoM/BoM: maturation in BoM/embryo culture in BOM (*n* = 103). The figure was prepared with BioRender.

The degree of cumulus cells expansion after 24 h of oocyte maturation using two different commercial media and with or without MSC addition was assessed as described by Lee et al. (16). The evaluation system was as follows: no expansion, limited expansion (less than three layers of cumulus cells expended), expended (more than three layers of cumulus cells expanded) and oocytes with no cumulus cells attached were classified as degenerated) (Figure 1).

#### Assessment of nuclear maturation

**Figure 2:**
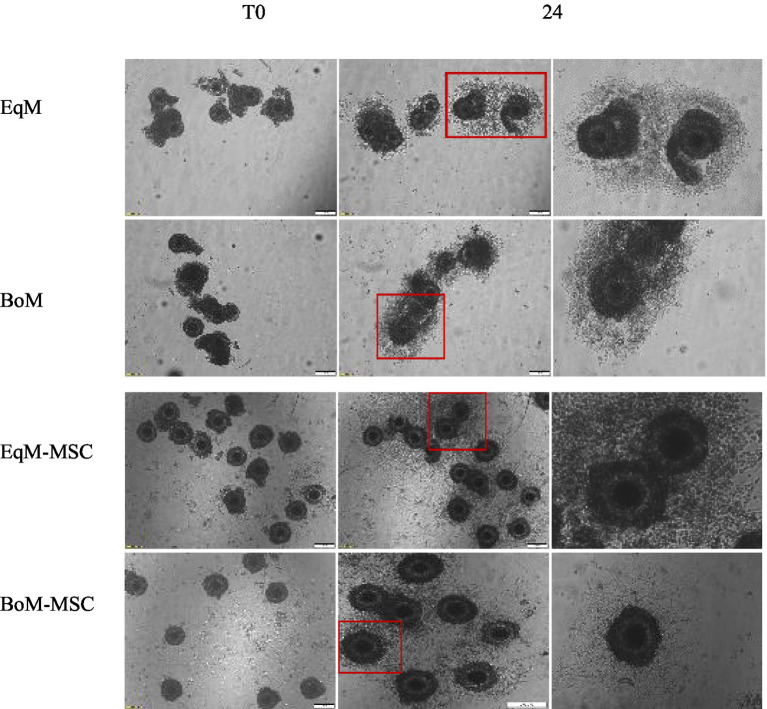
Effect of feline MSCs cells on cumulus expansion of oocytes after IVM. The degree of cumulus expansion at T0 and after 24 h of IVM in four groups EqM + MSC, BoM + MSC, EQM, and BoM. The images were taken with a magnification = 200 um.

The nuclear state of the stained oocytes was assessed under the fluorescence microscope (Olympus IX73) at excitation 360 and 450 nm emission. Oocytes with distinct polar bodies or two separate and bright chromatin spots were classified as entering the MII stage (Figure 2).

#### Experiment 2: the effect of the co-culture with MSCs during oocyte maturation on embryo development

This part of the study was done to assess the effect of the co-culture system with MSCs during maturation in two commercial media on the subsequent embryo development after *in vitro* fertilization. In total, 565 oocytes were matured and cultured in four groups,10 replicates per group, as illustrated in Figure 1. Embryonic development (cleavage, morula and blastocysts rate) was compared among all groups.

#### Experiment 3: the effect of co-culture with MSC during embryo development

At this stage, the oocytes were matured in EqM or in BoM then the MSCs were added during the embryo development to evaluate their effect on the morula and blastocyst formation. In total, 486 oocytes were matured and cultured in four groups, 10 replicates per group, as presented in Figure 1.

### Statistical analysis

Data were analysed using one-way ANOVA followed by Tukey’s multiple comparison test using Statistical software (TIBCO, United States). Values are shown as mean ± S.E.M. The significance level was *p* < 0.05, and at least three independent replicates were performed in all experiments. Nonparametric data, such as differences in the percentage values between groups, were assessed using the chi-square test.

## Results

### Effect of co-culture on the cumulus cells expansion

**Figure 3:**
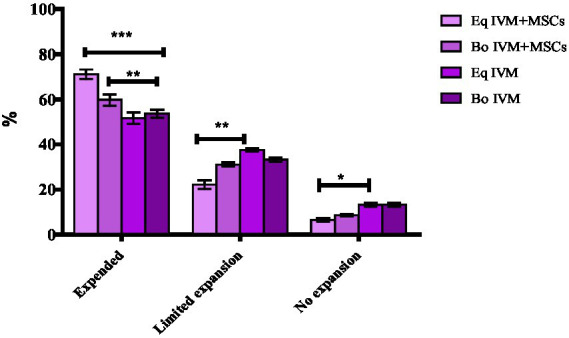
Effect of feline MSCs cells on cumulus expansion of oocytes after *in vitro* maturation. The cumulus expansion scoring system was as follows: limited expansion, no expansion, degenerated oocytes, denuded and expanded oocytes in four groups EqM + MSCs, BoM + MSCs, EQM, BoM. The Data are shown as the mean ± S.E.M. ^***^, ^**^, ^*^Within the columns, values are significantly different (*p* < 0.05).

The degree of COCs expansion was significantly increased in the EqM + MSCs and BoM + MSCs groups compared to EqM and BoM or groups (*p* < 0.05) (Figures 2, 3). Furthermore, there was no significant difference between the EqM + MSCs and BoM + MSCs groups. The evaluation of the degree of cumulus cell expansion confirmed that co-culture with MSC showed a considerable increase in the proportion of COCs that showed cumulus expansion (*p* < 0.05).

### Effect of different culture conditions on the efficacy of maturation of oocytes

**Figure 4:**
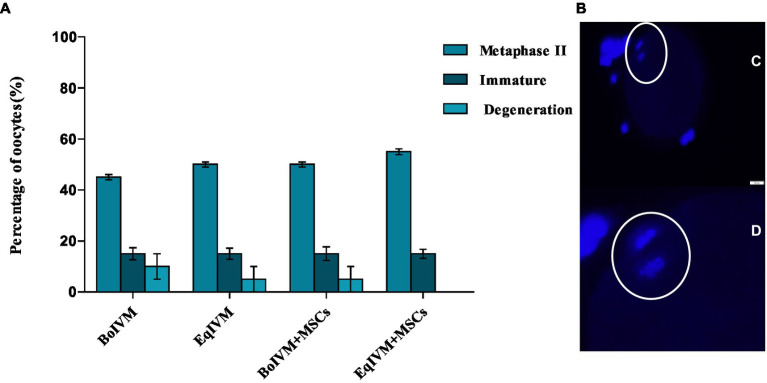
**(A)** Effect of different maturation conditions on nuclear maturation of feline oocytes. Metaphase II indicated oocyte maturation; the results are shown as the mean ± S.E.M. within the columns (*p* < 0.05). **(B)** Representative microscopy images of oocytes in metaphase II illustrating **(C, D)** polar body extrusion and nucleus stained with DAPI (Bar = 50 μm).

Nuclear maturation was evaluated using DAPI staining and showed a similar percentage of metaphase II (M II) (Figure 4B) in all investigated groups, which ranged from 45% to 55% (*p* > 0.05). The co-culture system with MSCs did not affect the nuclear maturation of oocytes (Figure 4A).

#### Development of embryos derived from oocytes matured under different maturation conditions

As shown in Table 1, the percentage of the oocytes which cleaved was similar in the co-culture group EqM + MSCs and BoM + MSCs and higher (*p* < 0.05) than in the EqM and BoM group. The rate of the morula was similar among EqM, BoM and BoM + MSCs groups but higher in the group of oocytes matured in EqM + MSCs. The oocytes matured in EqM + MSCs showed the most promising development and the highest number of blastocysts compared with the BoM + MSCs, BoM and EqM groups. Thus, the use of MSC as a co-culture during oocyte maturation has an effect on the further development of feline embryos (Table 1).

**Table 1:** Effect of different conditions during oocyte maturation on the subsequent embryo development.

Group	Oocytes number *n*, %	Cleavage *n*, %	Morula *n*, %	Blastocysts *n*, %
EqM + MSCs/EqM	109	78 (71.56)^a^	51 (46.79)^a^	28 (25.68)^a^
BoM + MSCs/BoM	124	84 (67.74)^c^	48 (38.71)^ab^	25 (20.16)^ab^
EqM/EqM	109	60 (55.04)^b^	40 (36.69)^ab^	18 (16.51)^b^
BoM/BoM	103	52 (50.48)^d^	35 (33.98)^b^	15 (14.56)^b^

EqM + MSCs/EqM, maturation in EqM + MSCs and embryo culture in EqM; BoM + MSCs/BoM, maturation in BoM + MSCs and embryos culture in BoM; EqM/EqM, maturation in EqM and embryo culture in EqM; BoM/BoM, maturation in BoM and embryo culture in BoM; (*p* < 0.05).

#### Development of embryos cultured under different conditions

The rate of development of the resulting two-cell embryos was higher in the co-culture group of EqM + MSCs and BoM + MSCs compared with two-cell embryos that were cultured in BoM and EqM (*p* > 0.05). However, we also noticed that the blastocyst/morula rate was higher in pure EqM media when compared to non-conditioned Bo culture media (*p* > 0.05).

## Discussion

*In vivo*, oocyte maturation occurs within the ovarian follicle, while fertilization and early embryo development occur in the fallopian tubes. When trying to recreate *in vitro* these physiological conditions, it is crucial to provide efficient culture systems (17); the first successful *in vitro* fertilization (IVF) in a domestic cat was achieved 45 years ago using *in vivo* matured oocytes and *in utero*-capacitated spermatozoa (18). Despite advances in culture conditions, media and protocols for oocyte maturation and embryo development, *in vitro* outcomes are still far from desirable compared with embryos produced *in vivo* (19, 20). In particular, recent studies have shown the beneficial effect of co-culture with MSCs (21), oviduct cells, and cumulus cells (22, 23) on the development of oocytes and embryos in various mammalian species, including cattle (24), horses (25), pigs (26) and canines (27). Therefore, culture conditions are crucial in determining the quality of *in vitro*-produced embryos. In the present study, we demonstrated for the first time the effect of feline Wharton’s jelly-derived MSCs and different commercial media on the maturation of feline oocytes, cumulus cell expansion and embryo development.

Oocytes with adequate nuclear and cytoplasmic maturation are more competent since many proteins and transcripts stored in their cytoplasm will be required for future embryo development. Therefore, in this study, we investigated whether the co-culture condition with MSCs as a feeder layer can influence oocyte maturation and their ability to develop into embryos. Based on the extrusion of the first polar body (metaphase II) in each experimental group, we did not observe the effect of co-culture with MSCs on the oocytes’ nuclear maturation resulting in comparable percentages ranging between 45 and 55% of MII. Similarly to our results, Ascari et al. (28) showed that murine MSCs or embryonic fibroblasts did not affect the nuclear maturation rate of bovine oocytes. In contrast, the addition of the conditioned medium containing human bone marrow MSCs, as a supplement to enrich the IVM medium used for germinal vesicles in mice polycystic ovary syndrome (PCOS) significantly increased cytoplasmic and nuclear maturation of oocytes (13).

However, when analysing the treatment used during maturation, we observe the morphological difference in cumulus cells expansion after 24 h of maturation; the oocytes cultured in MSCs-conditioned media had significantly increased the cumulus cell expansion compared to oocytes cultured without MSC addition. Similar observations were reported for human adipose-derived stem cells (ASC) added to the medium (ASC-CM) that improved cumulus cell expansion with high transcript abundance of an expansion-related gene in porcine (29). It was also reported by Wang et al. (30) that human Wharton’s jelly MSCs were used to treat mice with induced premature ovarian failure using a daily dose of intraperitoneal CTX injection (50 mg/kg) for 15 consecutive days; the results showed that MSCs reduced cumulus cell apoptosis in investigated mice. Other authors explored the use of human placental MSCs on human ovarian granulosa cells obtained from patients with premature ovarian insufficiency; the reported results showed that MSCs released epidermal growth factor (EGF) that reduced apoptosis and improved proliferation, and restored the oxidative enzyme levels of human granulosa and cumulus cells (31).

After fertilization, we observed differences between the oocytes that matured with and without MSCs. The embryos derived from the oocytes matured with MSCs: EqM + MSCs, BoM + MSCs showed a higher cleavage, morula, and blastocyst rate compared to the oocytes matured in classic BoM and EqM. We observed that the presence of MSCs during the maturation of oocytes did not affect nuclear maturation; it still affected the cleavage rate and blastocyst formation. As reported before, MSCs release several trophic factors, including EGF and cytokines. The trophic effects of these bioactive factors on preantral follicular growth and *in vitro* maturation of mouse oocytes have been shown (32); it was also reported that the conditioned medium containing human MSCs generated microenvironment that was more appropriate to induce oocyte maturation and increase embryo development of; they also described that high embryonic development rates might be associated with the quality of nuclear and cytoplasmic maturation (33).

The effect of co-culture with MSCs during embryo development was also evaluated in our study. In this part of the experiment, we noticed an improvement in embryo development (Table 2); the morula and blastocysts rate was higher in EqM + MSCs and BoM + MSCs than in BoM and EqM. It is interesting to point out that the embryos co-cultured with MSCs were previously maturated in classic BoM or EqM media. Our current findings are similar to the results of the previous study conducted by Jasmin et al. (34) using mice embryos; they observed that embryos co-cultured with MSCs for 4 days actually formed more blastocysts. Furthermore, our current data are similar to those shown by the same group using murine MSCs and embryonic fibroblast as a co-culture during embryo development (28).

**Table 2:** Effect of different culture conditions on the development of the embryos from the oocytes matured in BoM or EqM.

*Group*	Oocytes number *n*, %	Morula *n*, %	Blastocysts *n*, %
EqM/EqM	109	40 (36.69)^ab^	18 (16.51)^c^
BoM/BoM	103	35 (33.98)^b^	15 (14.56)^b^
EqM/EqM + MSCs	142	59 (41.54)^a^	26 (20.31)^a^
BoM/BoM + MSCs	132	52 (39.39)^ab^	22 (19.82)^abc^

EqM/MSCs + EqM, maturation in EqM and embryo culture in EqM + MSCs; BoM/BoM + MSCs, maturation in BoM and embryo culture in BoM + MSCs; EqM/EqM, maturation in EqM and embryo culture in EqM; BoM/BoM, maturation in BoM and embryo culture in BoM); (*p* > 0.05).

In general, the co-culture with somatic cells has shown a positive impact on embryonic development *in vitro.* Most studies indicate a higher rate of blastocyst formation after culturing embryos with different types of somatic cells (35, 36). However, some studies did not show significant improvement in embryo development (37, 38), and some others indicated a negative effect of the co-culture system on preimplantation embryo development (39). However, the co-culture studies published to date used very different types and concentrations of cells ranging from 1 × 10^3^ to 1 × 10^6^ cells/mL (24, 40), whereas our study used 1 × 10^4^ cells/mL, as a long with the main medium used, time points and oxygen concentrations, so comparisons are very hard and quite limited.

In fact, despite some similarities, each species may have different requirements regarding the substrates in the medium (41), which could explain the minor divergent results observed in our study. Here, we used the same source of mesenchymal cells, but we carried out the culture using two media dedicated to different species (cattle and horses). It is worth noticing that we noted more blastocysts with the use of equine media (EqM) compared to bovine (BoM). The latter could also have some impact on the results obtained during the co-culture experiment.

In summary, we investigated the potential of feline Wharton’s jelly MSC to assist in feline oocyte maturation and embryo growth. With the addition of feline Wharton jelly MSC both to oocyte and embryo culture, we observed an improved embryo development. Furthermore, our results did not show a significant impact on the nuclear maturation process itself, but the addition of MSCs as a feeder layer during the maturation or embryo culture still resulted in a higher rate of embryonic development. In particular, we found that the co-culture with MSCs was most effective during oocyte maturation, as the cleavage and blastocyst rates were higher when MSCs were added during oocytes maturation than during embryos development. These findings suggest that feline Wharton’s jelly MSCs could be a promising tool for improving *in vitro* feline embryo development in the future.

## Data availability statement

The original contributions presented in the study are included in the article/supplementary material, further inquiries can be directed to the corresponding authors.

## Ethics statement

Ethical approval was not sought, as it is not required for studies on cells obtained from surgical waste tissues (Decision No. 004/2021). The studies were conducted in accordance with the local legislation and institutional requirements. Written informed consent was obtained from the owners for the participation of their animals in this study.

## Author contributions

MO and MB: conceptualization, data collection, data evaluation, statistical analysis, and manuscript writing. PM: data evaluation and reviewing of the paper. YL: reviewing and editing the paper. WN: consulting and supervising the study and paper reviewing. All authors contributed to the article and approved the submitted version.
